# Evaluating the Implementation of a “COVID-19 Test” Chief Concern in the Emergency Department

**DOI:** 10.5811/westjem.34850

**Published:** 2025-05-02

**Authors:** Collin Michels, Daniel J. Hekman, Rebecca J. Schwei, Ryan E. Tsuchida, Joshua Gauger, Irene Hurst, Joshua Glazer, Jenna Brink, Ciara Barclay-Buchanan, Manish N. Shah, Azita G. Hamedani, Michael Pulia

**Affiliations:** * University of Wisconsin–Madison School of Medicine and Public Health, BerbeeWalsh, Department of Emergency Medicine, Madison, Wisconsin; † University of Wisconsin–Madison, College of Engineering, Industrial and Systems Engineering, Madison, Wisconsin

## Abstract

**Background:**

During the COVID-19 pandemic, rapid, at-home testing for severe acute respiratory syndrome-coronavirus-2 (SARS-CoV-2) was inconsistently available. Consequently, for some patients, emergency departments (ED) became the preferred site to access COVID-19 testing. To improve operational efficiency, our ED implemented a “COVID-19 Test” chief concern (CC). Our primary objective in this analysis was to broadly assess the utilization of the new “COVID-19 Test” CC and associated clinical care.

**Methods:**

We conducted a retrospective analysis of ED encounters from an academic ED and an affiliated, community-based ED of all patients after the establishment of a CC of “COVID-19 Test” from October 11, 2021–July 31, 2022. The data were extracted from the electronic health record. We calculated descriptive demographic statistics and ran a univariate and multivariate logistic regression with additional diagnostic or therapeutic interventions (binary) as the outcome variable to generate odds ratios (OR) and 95% confidence intervals (CI).

**Results:**

A total of 320 patients were assigned a “COVID-19 Test” CC by a triage nurse. This was 0.5% of all ED encounters in this time frame. Of those, 45% were found to be SARS-CoV-2 positive. Admission or repeat ED visit at 72 hours occurred in 5.3% of patients. Nearly half (46.9%) of patients assigned a “COVID-19 Test” CC underwent additional ED interventions. Patients on Medicaid and those who self-identified as Black or Hispanic/Latino were disproportionately represented in the “COVID-19 Test” CC group as compared to the overall ED population. In multivariate analysis, an Emergency Severity Index of 1, 2 or 3 was associated with significantly higher odds of receiving additional interventions compared to ESI of 4 or 5 (adjusted OR: 46.85; 95% CI 13.28–165.26; *P* <0.001).

**Conclusion:**

Patients assigned a chief concern of “COVID-19 Test” had a high COVID-19 positivity rate, often underwent additional ED interventions, and were at low risk of return ED visits or admission. Minoritized and low-income patients were disproportionately represented in the “COVID-19 Test” CC group, highlighting potential disparities in access to at-home COVID-19 testing and implementation of this CC.

## INTRODUCTION

Throughout the various phases of the COVID-19 pandemic, access to diagnostic testing evolved significantly. Even after rapid at-home tests were eventually approved, they were not always readily available for purchase.^1^ Although insurance companies were required to cover the cost of eight tests per month during the public health emergency, this involved a burdensome reimbursement process.^1^ Additionally, those without insurance had to rely on tests distributed by the federal government or pay out of pocket for these expensive tests.^1^ Therefore, emergency departments (ED) became a site to access COVID-19 testing for some patients throughout the pandemic.

We implemented an operational intervention to rapidly identify and expedite care for patients presenting to the ED for the reported sole purpose of receiving COVID-19 testing. Better understanding of the characteristics and clinical course of patients who visit the ED only to receive a COVID-19 test could inform policies and procedures for future surges of COVID-19 and other respiratory pathogens. The primary objective of this analysis was to assess the uptake of this operational intervention and its performance in identifying the target population. Our secondary objective was to describe the demographic and clinical course of the ED patients assigned a chief concern (CC) of “COVID-19 Test,” including a comparison between those who subsequently were discharged after solely receiving COVID-19 testing vs those who received additional diagnostic testing or therapeutics.

## METHODS

### Setting and Intervention

On October 11, 2021, a health system in the Midwestern United States, a quaternary-care academic ED that sees approximately 60,000 visits per year and a community hospital-based ED with approximately 24,000 visits annually, added “COVID-19 Test” to the list of CC options eligible for selection by the ED triage nurse or designee. The CC selected is meant to identify the patient’s primary reason for the ED visit. Triage nurses were informed of the addition of this CC and educated on its intended use prior to rollout at daily nursing huddles that occurred before each new shift. This quality improvement initiative assessment was deemed exempt based on institutional review board criteria, and informed consent was waived.

### Data Source

We conducted a retrospective analysis of ED patient encounters from October 11, 2021–July 31, 2022 that had “COVID-19 Test” assigned as the CC and received a COVID-19 polymerase chain reaction test to detect severe respiratory syndrome coronavirus-2 (SARS-COV-2). Ending data collection in July allowed us to capture the first full respiratory season after the CC was implemented. Structured healthcare data were extracted from the electronic health record (Epic, Verona, WI) by DH, a department data analyst. We divided the sample into two groups: 1) COVID ONLY, for those who received only COVID-19 testing; and 2) COVID PLUS, for those patients who received additional diagnostic testing (any laboratory or imaging studies) or therapeutics (any medications or procedures).

**Population Health Research Capsule**

What do we already know about this issue?

*Early in the COVID-19 pandemic, reliable at-home testing was not widely available and EDs provided testing and developed new operational workflows.*

What was the research question?

*We sought to broadly assess the use of a “COVID-19 Test” chief concern and associated clinical care.*

What was the major finding of the study?

*Patients with this chief concern were disproportionally Black or Hispanic, and 45% tested positive. Emergency Severity Index 1–3 patients had higher odds of additional interventions (aOR: 43.28; 95% CI 12.45–150.43; P <0.001).*

How does this improve population health?

*Understanding impacts of assigning chief concerns in the ED can inform future operations and health disparities research.*

We extracted information on patient demographics including age; age group (pediatric [<18], adult [18–64], and older adult [≥65]); sex (male or female); patient-reported race (American Indian or Alaska Native, Asian, Black, Native Hawaiian or Pacific Islander, White, or patient declined to answer); patient-reported Hispanic, Latino or Spanish ethnicity (yes, no, declined to answer); and insurance status (commercial, Medicaid, Medicare, or other (self-pay, workers compensation, or unknown insurance). We extracted information about the ED visit including disposition (admitted, discharged, left before treatment complete); COVID 19 test result (positive, negative); and Emergency Severity Index triage score (ESI). The ESI is meant to reflect a combination of patient acuity and intensity of resource requirement. It is assigned on a scale of 1–5, with one usually reserved for patients in need of emergent resuscitation and five usually indicating non-urgent patient presentation. Vitals sign data was captured using triage vitals, including temperature, heart rate, oxygen saturation, respiratory rate, and systolic and diastolic blood pressure. We classified patients as having abnormal temperature, heart rate, oxygen saturation, respiratory rate, and systolic or diastolic blood pressure, if the patient had a temperature >100.4°F; heart rate or pulse >100 beats per minute; oxygen saturation 92%; respirations >24 breaths per minute; systolic blood pressure <100 or >200 millimeters of mercury (mmHg); diastolic blood pressure >100 mmHg, respectively. We generated a summary variable that indicated whether the patient had any abnormal vital sign (yes/no). We also recorded whether participants had a return ED visit within 72 hours of initial ED discharge.

### Analysis

We compared differences in demographic and clinical encounter variables between the COVID ONLY and COVID PLUS groups, using *t*-tests for continuous variables and independent chi-square test for categorical variables. We also compared demographic characteristics between the patient cohort flagged with the “COVID-19 Test” CC vs the entire ED population. We built a multivariable logistic regression model to describe the odds of receiving only a COVID test according to patient demographic and clinical encounter variables. We included variables in the model that prior literature (race, ethnicity, insurance status, age)^2^–^4^ or clinical factors (COVID test result, ESI) suggested might be related to differences in ED utilization or care patterns. We excluded from the multivariate analysis 26 patients with missing values and seven patients who declined to answer demographic questions. Due to small numbers, the race ethnicity variable was included in the model as a dichotomous variable (White and non-Hispanic, Latino or Spanish origin; or non-White and/or Hispanic, Latino or Spanish origin). Alpha of 0.05 was prespecified as the threshold of statistical significance for all tests. We performed all data retrieval, cleaning, and analysis using R 3.6.3 (R Foundation for Statistical Computing, Vienna, Austria).^5^

## RESULTS

A total of 320 patients were assigned a CC of “COVID-19 Test,” which represented 0.5% of the patient population during this period. Of those patients with a CC of “COVID-19 Test,” 45.0% tested positive for COVID-19 (Table 1). The average age was 30.5 years old with a standard deviation of 19.6 years; 26.2% of patients were pediatric; and 6.9% were older adults. Overall, 39.7% of patients with a CC of “COVID-19 Test” identified as Black and 10.3% as Hispanic/Latino compared with the overall ED population from the study period of 11.8% and 7.9%, respectively. In terms of insurance status, 51.9% of “COVID-19 Test” CC patients were covered by Medicaid insurance compared with 22.0% of the general ED patient population. In the “COVID-19 Test” group, 2.2% of patients were admitted compared to 25% of the general ED population and 3.1% of patients had a return ED visit within 72 hours as compared to 0.9% in the general ED population. Use of the “COVID-19 Test” CC was highest in December 2021 and January 2022 (27.5% [88/320], and 35.6% [114/320]), respectively. The other eight months of the intervention had utilization that ranged from eight uses of the CC in March of 2022 to 25 uses in November 2021. This pattern corresponded to community rates of influenza-like illness, which surged in the winter of 2021–2022.

Approximately half (53.1%) of patients with a “COVID-19 Test” CC only received a PCR test for SARS-CoV-2 (COVID ONLY group), while the other 46.9% had PCR testing for SARS-CoV-2 plus additional ED interventions (COVID PLUS group). Within the COVID Plus group 74.7% (112/150) of patients had additional diagnostics and 70.7% (106/150) of patients were given additional therapeutics. Within therapeutics, 94.3% (100/106) of patients received at least one medication. Compared to the COVID PLUS group, the COVID ONLY group was significantly younger; more likely to identify as Black; more likely to identify as Hispanic or Latino, less likely to have Medicare; less likely to be admitted; less likely to have tested positive for COVID-19; less likely to have an abnormal vital sign; and more likely to have ESI scores of 4 or 5 (Table 1).

In the multivariable analysis, non-White patients or patients with Hispanic, Latino or Spanish ethnicity had 0.52 decreased odds of receiving additional interventions compared to White patients with non-Hispanic, Latino or Spanish ethnicity (95% confidence interval [CI] 0.29–0.93; P=0.03). Additionally, patients with Medicaid for insurance had 2.57 increased odds of receiving additional testing compared to privately insured patients (95% CI 1.08–3.98; P = 0.03). Patients who tested negative for COVID-19 had a statistically significant decreased odds of receiving additional interventions (adjusted odds ratio (aOR) 0.57; 95% CI 0.33–0.99; *P*=0.05) while controlling for other demographic and severity covariates (Table 2). Patients with an ESI of 1, 2 or 3 had significantly higher odds of receiving additional interventions compared to patients with an ESI of 4 or 5 (aOR 43.28; 95% CI 12.45–150.43; *P* <0.001) when controlling for age group, sex, race, ethnicity, insurance, and COVID-19 result.

## DISCUSSION

This analysis uniquely evaluates the outcome of an operational intervention to identify patients presenting to the ED, reportedly, for the sole purpose of accessing COVID-19 testing. Post hoc analyses of operational improvement initiatives are critical in assessing impact and identifying unintended consequences. By embedding race and ethnicity in this process evaluation, our analysis aligns with the recently published quality framework to address racial and ethnic disparities in ED care.^6^

The “COVID-19 Test” CC did not consistently identify patients who would only need a SARS-CoV-2 PCR vs those whose presentation necessitated greater ED management, as nearly half of the patients assigned this CC received additional diagnostic evaluation or therapeutic interventions. Additionally, an ESI score 1, 2 or 3 and ≥1 abnormal vitals were present in approximately 1 of 4 patients, which suggests that many patients were determined by the treating clinician to require additional ED evaluation beyond COVID-19 testing based on triage ESI and vital signs. Given the ongoing national boarding crisis, expected seasonal resurgences in COVID-19, and seasonal influenza along with other infectious disease outbreaks, the ability to rapidly identify low-acuity patients presenting to the ED for infectious disease testing only will be an increasingly important operational goal. One important refinement to this operational intervention would be to do additional training of triage staff on optimal assignment and/or limiting its use only to patients with the lowest acuity triage scores (ESI 4 and 5).

The COVID-19 positive rate for the study population was over four-fold higher than the reported community positivity rates (45% vs 10%).^7^ This implies that these patients did not view the ED as a simple alternative to testing at home or at another clinical site. Rather, sufficient barriers to using alternative testing sites exist for this group such that they generally present to the ED when they feel sufficiently ill or perceive a higher likelihood of COVID-19 (eg, close contact with confirmed infection). The high positivity rate indicates that anyone identified in triage as reportedly seeking only COVID-19 testing should be presumed positive until proven otherwise as it pertains to infection control measures. Thus, while the operational intervention failed to identify a low-acuity, low-resource-requiring patient subgroup, it did identify a group of patients at substantially greater risk for infection than the general population. Grouping patients into high, intermediate or low-risk groups has been suggested previously^8^ and may still be a worthy outcome to pursue in future COVID-19 surges.

While there was a high positivity rate in this sample, over half of the patients (55%) tested negative for COVID-19. Among the patients who tested negative, three of five received no additional interventions. These findings do indicate that for some patients the ED may have served as a place to get COVID-19 testing. This suggests that there is utility in refining operational interventions such as this one to identify low-acuity patients.

Regarding the study’s secondary objective, we observed differences in racial and ethnic demographics and care patterns in patients assigned the “COVID-19 Test” CC. Specifically, patients identifying as Black were represented in the study group at 3.5 times the general ED population. Patients with Medicaid insurance were represented twice as much in this study population compared to the general ED population. Such representation of Medicaid patients in our study population is in line with a recent Agency for Healthcare Research and Quality report noting higher rates of ED visits due to COVID-19 among poor or near-poor individuals.^9^ This raises important concerns about barriers to reliable COVID-19 testing and non-ED care for both minoritized and low-income patient groups, for which there is substantial demographic overlap.^10^ Our findings found a similar unequal availability of COVID-19 testing as described in New York City.^11^ The disproportionate representation of Medicaid patients assigned this CC indicates a potential missed opportunity to improve access to on-demand COVID-19 testing in non-ED settings among this managed population.

Patients who identified as White were more likely to undergo additional diagnostic testing compared to non-White ethnicities. This is consistent with previous studies that have demonstrated disparities in ED testing and treatment in these groups including for acute pain management^12^ and long bone fractures.^13^ Additionally, the ESI score functioned as intended and drove additional interventions. Specifically, the COVID ONLY subgroup had only three patients (1.8%) assigned ESI 1, 2, or 3 compared to 73 patients (48.6%) in the COVID PLUS group. Thus, any intentional or unintentional under-triage of minority and marginalized populations would substantially alter the access to clinical care and resources for this population.^14^ Recent work by Essa et al observed cognitive bias in ESI score assignment across all levels of severity, which were most pronounced for lower acuity patients (ESI 3–5).^15^ This marks a potential limitation for our study and underscores the need to consider de-biasing strategies when implementing new care pathways initiated in triage.

## LIMITATIONS

This was a retrospective analysis, and it is possible that unmeasured variables might have contributed to our observations. Further, this project was conducted in a single healthcare system, and the results may not be generalizable to other settings. Finally, due to small sample sizes we were not able to compare outcomes between sites.

## CONCLUSION

A retrospective analysis of patients assigned a “COVID-19 Test” chief concern demonstrated that these patients had a high COVID-19 positivity rate and often underwent additional ED interventions. Demographic differences between the overall ED population and those assigned the “COVID-19 Test” CC suggest patients from historically marginalized and low-income groups may more likely be classified under this CC and/or experience disproportionate barriers to accessing COVID-19 testing. Differential ED interventions for these groups also highlight the need for further consideration of disparities related to COVID-19 evaluation and treatment in the ED.

## Figures and Tables

**Table 1:** Description of patient demographic and clinical encounter variables overall and by COVID-19 lab only and additional intervention group, n (%).

	Overall (N=320)	COVID Only (n=170)	COVID Plus (n=150)	*P*-value*
Age (mean [SD])	30.5 (19.6)	25.3 (17.2)	36.5 (20.5)	<0.001
Age Group				<0.001
Pediatric (0–17)	84 (26.2)	59 (34.7)	25 (16.7)	
Adult (18–64)	214 (66.9)	107 (62.9)	107 (71.3)	
Older Adult (65+)	22 (6.9)	4 (2.4)	18 (12.0)	
Male	163 (47.1)	84 (49.4)	68 (45.3)	0.54
Race				0.01
American Indian or Alaska Native	9 (2.8)	6 (3.5)	3 (2.0)	
Asian	16 (5.0)	11 (6.5)	5 (3.3)	
Black	127 (39.7)	77 (45.3)	50 (33.3)	
Native Hawaiian or Pacific Islander	0 (0.0)	0 (0.0)	0 (0.0)	
White	161 (50.3)	70 (40.1)	91 (60.7)	
Patient declined to answer	7 (2.2)	6 (3.5)	2 (0.7)	
Patient Reported Ethnicity				0.02
Hispanic/Latino	33 (10.3)	24 (14.1)	9 (6.0)	
Not Hispanic or Latino	285 (89.1)	144 (84.7)	141 (94.0)	
Patient declined to answer	2 (0.6)	2 (1.2)	0 (0.0)	
Insurance Status				0.01
Commercial	107 (33.4)	64 (37.6)	43 (28.7)	
Medicaid	166 (51.9)	91 (53.5)	75 (50.0)	
Medicare	34 (10.6)	9 (5.3)	25 (16.7)	
Other coverage†	13 (4.1)	6 (3.5)	7 (4.7)	
Disposition				0.01
Admit	7 (2.2)	0 (0.0)	7 (4.7)	
Discharge	307 (95.9)	165 (97.1)	142 (94.7)	
Left before treatment complete	6 (1.9)	5 (2.9)	1 (0.7)	
COVID-19 Test Result				0.01
Positive	144 (45.0)	64 (37.6)	80 (53.3)	
Negative	176 (55.0)	106 (62.4)	70 (46.7)	
Emergency Severity Index				<0.001
1	0 (0.0)	0 (0.0)	0 (0.0)	
2	8 (2.5)	0 (0.0)	8 (5.3)	
3	68 (21.2)	3 (1.8)	65 (43.3)	
4	182 (56.9)	112 (65.9)	70 (46.7)	
5	62 (19.4)	55 (32.4)	7 (4.7)	
Any abnormal vital signs	87 (27.2)	37 (21.8)	50 (30.3)	0.03
Return ED visit within 72 hours	10 (3.1)	3 (1.8)	7 (4.7)	0.24

*COVID-19*, coronavirus 2019.

* Comparison of group that had COVID-19 test only vs. additional workup, using chi-square to test differences across categorical variables and *t*-test for differences in mean for age.

† Other coverage included self-pay, workers’ compensation, and other insurance.

**Table 2:** Unadjusted and adjusted odds of receiving additional interventions by demographic and clinical encounter variables (n=313).

	Unadjusted Odds Ratio	Adjusted Odds Ratio	95% Confidence Interval; *P*- value
Age (years)			
<18	0.42*	0.63	0.33 – 1.29; 0.15
18 to 65	ref	ref	ref
≥65	4.50*	0.49	0.08 – 3.15; 0.45
Male	0.85	0.98	0.56 – 1.71; 0.94
Non-White race or LatinX ethnicity	0.36**	0.52*	0.29–0.93; 0.03
Insurance			
Commercial	ref	ref	ref
Medicaid	1.23	2.08*	1.08–3.98; 0.03
Medicare	4.13*	2.57	0.74 – 8.91; 0.14
Other Coverage	1..74	2.63	0.65 – 10.66; 0.18
COVID-19 negative	0.53	0.57*	0.33 – 0.99; 0.05
ESI Level			
1, 2 or 3	52.77**	43.28**	12.45 – 150.43; <0.001
4 or 5	ref	ref	ref

*COVID-19*, coronavirus 2019; *ESI*, Emergency Severity Index triage score.

* *P* <0.05;

** *P* <0.001
